# Molecular Phenotypes of Sex- and Age-Specific Differences Relating to Outcome in Patients With Ebola Virus Disease

**DOI:** 10.1093/infdis/jiag305

**Published:** 2026-06-17

**Authors:** Xiaofeng Dong, Natasha Y Rickett, Piet Maes, Robert Orr, Tracy MacGill, Todd Myers, Tom Fletcher, Miles W Carroll, Julian A Hiscox

**Affiliations:** Institute of Infection, Veterinary and Ecological Science, Faculty of Health and Life Science, University of Liverpool, UK; Institute of Infection, Veterinary and Ecological Science, Faculty of Health and Life Science, University of Liverpool, UK; European Plotkin Institute for Vaccinology, Universite Libré de Bruxelles (ULB), Belgium; Office Regulatory and Emerging Science, Office of the Chief Scientist, US Food and Drug Administration, Silver Spring, Maryland, USA; Office Regulatory and Emerging Science, Office of the Chief Scientist, US Food and Drug Administration, Silver Spring, Maryland, USA; Office Regulatory and Emerging Science, Office of the Chief Scientist, US Food and Drug Administration, Silver Spring, Maryland, USA; Department of Clinical Sciences, Liverpool School of Tropical Medicine, Liverpool, UK; The Pandemic Institute, Liverpool, UK; Wellcome Centre for Human Genetics; Pandemic Sciences Institute, Nuffield Department of Medicine, University of Oxford, Oxford, UK; Institute of Infection, Veterinary and Ecological Science, Faculty of Health and Life Science, University of Liverpool, UK; The Pandemic Institute, Liverpool, UK; A*STAR Infectious Diseases Labs, A*STAR, Singapore

**Keywords:** age, Ebola virus, Ebola virus disease, hemorrhagic fever, RNA-seq, sex, transcriptomics

## Abstract

**Background:**

Ebola virus causes a severe, potentially fatal disease with the outcome likely related to viral and host factors. There is a clear correlation between viral load and outcome (survival/death), and inflammatory and other cellular pathways are differentially activated in the acute stage between patients who survive and those who die. Age and sex may influence the course of Ebola virus disease with potential differences at the molecular level.

**Methods:**

To investigate whether there were age- and/or sex-specific differences in patients with Ebola virus disease, previously published data from an RNA sequencing analysis of the blood transcriptome in patients from the 2013–2016 West African outbreak were stratified according to age and sex. This was correlated with the outcome of infection.

**Results:**

Similar to the previously published data, immune responses were downregulated during the acute phase in hospitalized survivors as compared with hospitalized fatal cases, regardless of the sex of the patient. However, aspects of the blood transcriptome/host response differed between females and males and certain age groups at the transcriptomic level. Genes associated with the lymphocyte differentiation pathway decreased in expression level with age in hospitalized fatal cases. However, this was the opposite in hospitalized survivors.

**Conclusions:**

From a medical countermeasure perspective, the work indicated that selection of animal models should consider age and sex, depending on the research question, and cellular pathways were identified that could be chemotherapeutically enhanced to promote survival.

## Teaser

We analyzed blood transcriptomic data from West African patients who were acutely ill with Ebola virus disease who survived or died. The results showed sex-based differences in viral genetics and transcriptomes and age-related variations in host immune responses at the transcriptomic level.

The interplay between Ebola virus disease (EVD) and outcome is complex and likely involves a balance between the host immune response and viral load. Higher Ebola virus (EBOV) loads have been associated with severe disease and fatal infection in human disease and animal models of infection [1–4]. Several biomarkers of the host immune response have been associated with immunopathology and correlated with patient outcome (ie, fatality/survival). These include the strength of interferon signaling, the acute phase response, creatinine levels, NK-cell populations, and T-cell responses [5–9].

Age and/or sex can influence the host response and patient outcome in viral infections. Here, sex is defined as referring to the biological characteristics of females and males. Findings from the European Mobile Laboratory (EMLab) during the West African EVD outbreak indicated a relationship between age and case fatality rate (CFR) [4]. The CFR associated with age began to increase in patients within the 60- to 64-year age bracket. These age patterns were generally reflected in other epidemiologic studies [10–12]. The data from the EMLab showed no sex-specific bias in outcome [4]. Analysis of data from Guinea, Liberia, Nigeria, and Sierra Leone indicated that the average time from onset of symptoms to hospitalization was slightly less in females and that females were slightly more likely to survive than males, with respective CFRs of 63% and 67.1% [13]. The World Health Organization Ebola response team suggested no statistically significant difference in the percentage of cases between females and males, but the fatality rate was significantly higher for males once infected [13].

Given the potential differences in outcome between females and males with EVD, the blood transcriptomes of patients acutely ill with EVD were characterized to investigate whether there were sex-specific differences. Likewise, viral population genetics was also characterized, as variation in this may influence viral load and patient outcome [14]. Several sex-specific differences in the EVD-associated blood transcriptome were identified. Interestingly, the lymphocyte differentiation pathway was downregulated in older patients with EVD, highlighting that immunomodulatory strategies may enhance survival in certain cohorts of patients.

## METHODS

### Sample Collection, Sequencing, and Data Collection

Sequencing data used in this project were obtained from individual blood samples taken from the patients by the EMLab as part of the global response to the EVD outbreak in West Africa between 2013 and 2016. The sample IDs for the data used in this analysis, patient outcomes for the patient and EBOV viral loads (cycle threshold [Ct]) are summarised in Supplementary Table 1, and the sequencing approach has been described previously [5].

### Differential Expression Analysis

Hisat2 version 2.1.0 [15] was used to map the trimmed reads on the human reference genome assembly GRCh38 with known splice sites (release 91) downloaded from the Ensembl FTP site. The alignments were used for calculating read counts per gene via featureCounts version 2.0.0 [16] with a guide Ensembl GTF (release 91). The raw counts generated from featureCounts were imported into the R version 4.3.1 environment to carry out differential expression analysis via edgeR version 4.0.16. Differentially expressed genes (DGEs) with a false discovery rate <0.05 and an absolute log_2_ fold change >1 were reported. A false discovery rate–adjusted *P* value (also called *q* value) of .05 indicates that 5% of significant tests will result in false positives. Gene Ontology (GO) enrichment in the biological process for the DGEs was performed using the enrichGO function in clusterProfiler version 4.10.1 [17] to query the human GO database in org.Hs.eg.db version 3.14.0. The emapplot function in clusterProfiler was used to organize the enriched GO terms into an enrichment map based on the Jaccard index calculated by the pairwise_termsim function in clusterProfiler.

### Consensus Virus Genomes and Viral Nucleotide and Amino Acid Changes

The reads that were unmapped to the human reference genome assembly were extracted by bam2fastq (version 1.1.0). AioMinor was used to call the consensus virus genomes and corresponding nucleotide/amino acid sequences from the extracted reads, using a known EBOV genome as the reference (GenBank sequence accession KY426690) [18]. AioMinor was run with the default settings, while duplicate reads with a mapping quality score <11 were discarded. As a result, a consensus genome sequence of EBOV was generated for each hospitalized fatal case and survival case. The site nucleotide/amino acid variation frequency generated by AioMinor is the ratio of the nucleotide/amino acid deviation number to the read coverage at each site of EBOV genome sequences.

### Correlation Analysis

Correlation analysis was used to examine the strength and direction of the relationship between 2 variables. The analyses determined whether changes in one variable were associated with changes in another, without implying causation [19]. In the correlation analyses of “age versus 1/Ct” and “sex versus 1/Ct,” “age” and “1/Ct” were continuous variables whereas “sex” was a binary variable. After the normality of the continuous variables was assessed via the Shapiro-Wilk *W* test, the data for “age” and “1/Ct” did not follow a normal distribution. Therefore, the Spearman correlation method wvas used for both analyses (0 = male, 1 = female), where a correlation coefficient of −1 indicated a strong negative correlation and +1 indicated a strong positive correlation.

### Weighted Correlation Network Analysis

Gene network analysis was performed as previously described [20, 21]. The input normalized count was prepared using rlog functions with option “blind = TRUE” in the DESeq2 version 1.42.1 package. All sequenced samples (Supplementary Table 1) were used for weighted correlation network analysis (WGCNA), while the outgroups identified by R function hclust in WGCNA package version 1.73 [22] were excluded in the downstream analysis. Then modules were derived from the WGCNA package with the blockwiseConsensusModules function (power = 8 and 4, TOMType=“signed,” corType=“bicor,” maxPOutliers=0.05, minModuleSize=30, deepSplit = 4). Hub genes in these modules were calculated by the chooseTopHubInEachModule function from the WGCNA package.

## RESULTS

To investigate potential sex-specific differences in EVD outcomes, data from diagnostic leftover blood samples [5] were reanalyzed considering the sex and age of the patient. Transcriptomic data from sequencing the blood samples were derived from a hospitalized survivor group (n = 49) and a hospitalized fatal group (n = 84). The EBOV genomes in these hospitalized survivors and fatal cases were further analyzed if the final assembled dominant genome sequence was >18 800 nucleotides and contained no gaps. The samples used in this study are summarized in Supplementary Table 1. These samples were categorized and analyzed as shown in Supplementary Figure 1.

### Correlation Between Viral Load and Sex/Age

The correlation between EBOV Ct values (used henceforth as a proxy for viral load) and age and between EBOV Ct values and sex was tested by the Spearman rank correlation coefficient. EBOV Ct values were not significantly correlated with either age or sex in the patient samples analyzed. This suggested that the viral load did not vary with age or sex in the patients included in this study (Supplementary Figure 2). Therefore, viral load was not used as a variable in the remaining analyses.

### Sex- and Age-Specific Differences in Host Blood Transcriptomes

To explore the impact of age on outcome of EVD in humans, the hospitalized survivors and fatal cases were categorized into 3 age groups: 0 to 4 years (n = 8), 5 to 45 (n = 93), and >45 (n = 32) based on the concept of immunologic age (hereafter, 0–4, 5–45, and >45 groups) [23]. Within each age group, the fold change pattern of hospitalized survivors to fatal cases was compared between female and male patients. In the 5–45 group, more genes had similar expression levels between hospitalized survivors and fatal cases as compared with the 0–4 and >45 groups, regardless of the sex of the patient (Figure 1). In contrast, among those aged 0–4 years, 1 surviving female patient had fewer genes with significant fold changes (vs 3 hospitalized female survivors) when compared with 1 male survivor (vs 3 hospitalized male survivors).

**Figure 1.**
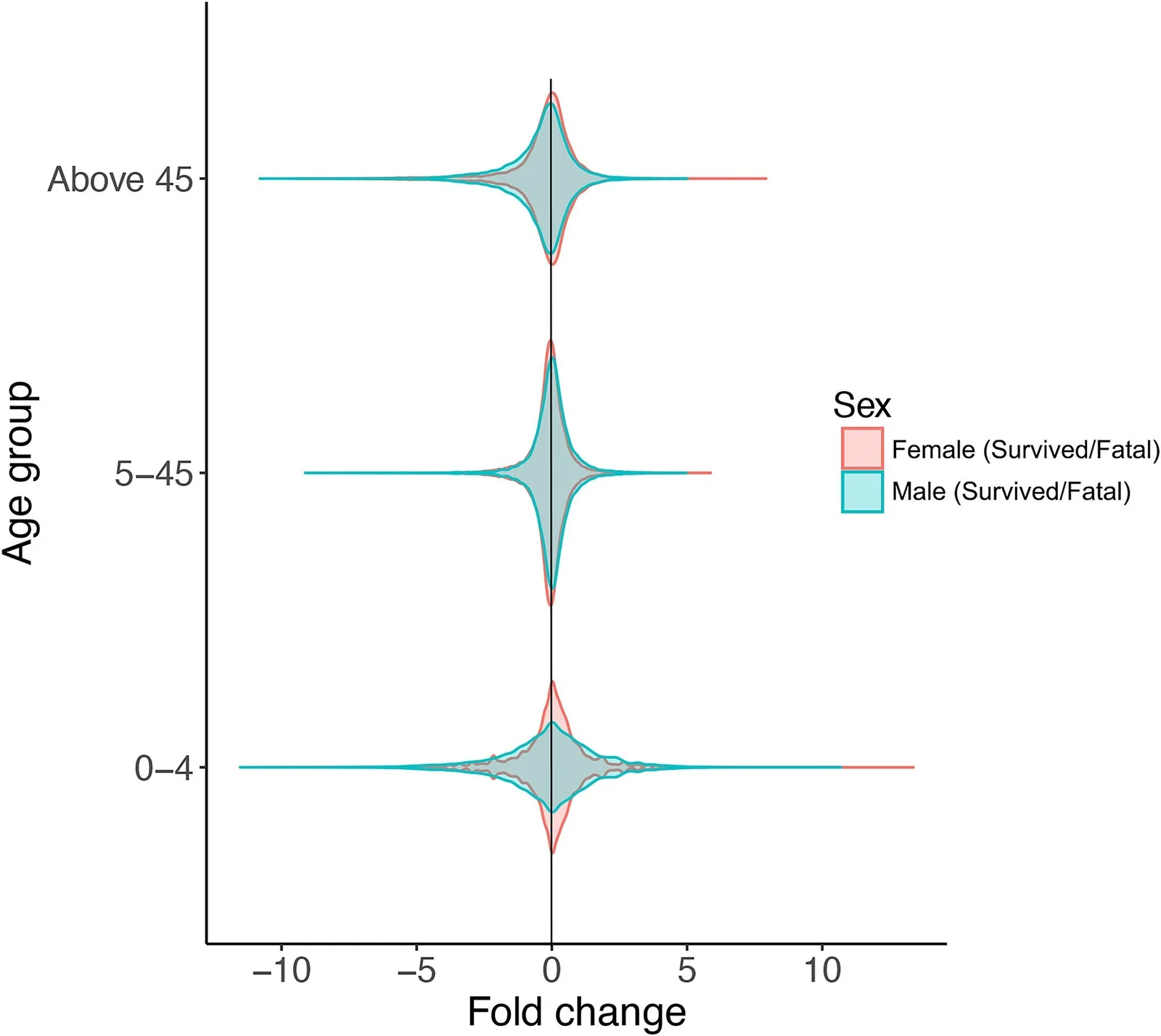
The fold change of overall gene expression levels of hospitalized survivors and fatal cases in female (red) and male (blue) patients. The height of the violin plot corresponds to the number of genes with the same fold change. Genes shown on the vertical line at fold change 0 indicate expression levels that are same in hospitalized survivors and fatal cases.

Following the comparison of overall gene expression patterns (Figure 1), significant DGEs were identified with edgeR. Without considering the sex of the patient, GO enrichment analysis revealed that the downregulated genes in hospitalized survivors were significantly enriched in leukocyte migration and wound healing (Figure 2). Most of these enriched GO terms are specific and were further clustered by the Jaccard index, which measures the similarity between 2 datasets (Supplementary Table 2, Supplementary Figure 3). Major clusters were related to cellular migration and wound-healing pathways in the 5–45 and >45 groups, but no specific enrichment clusters were identified in the 0–4 group (Figure 2, Supplementary Figure 3).

**Figure 2.**
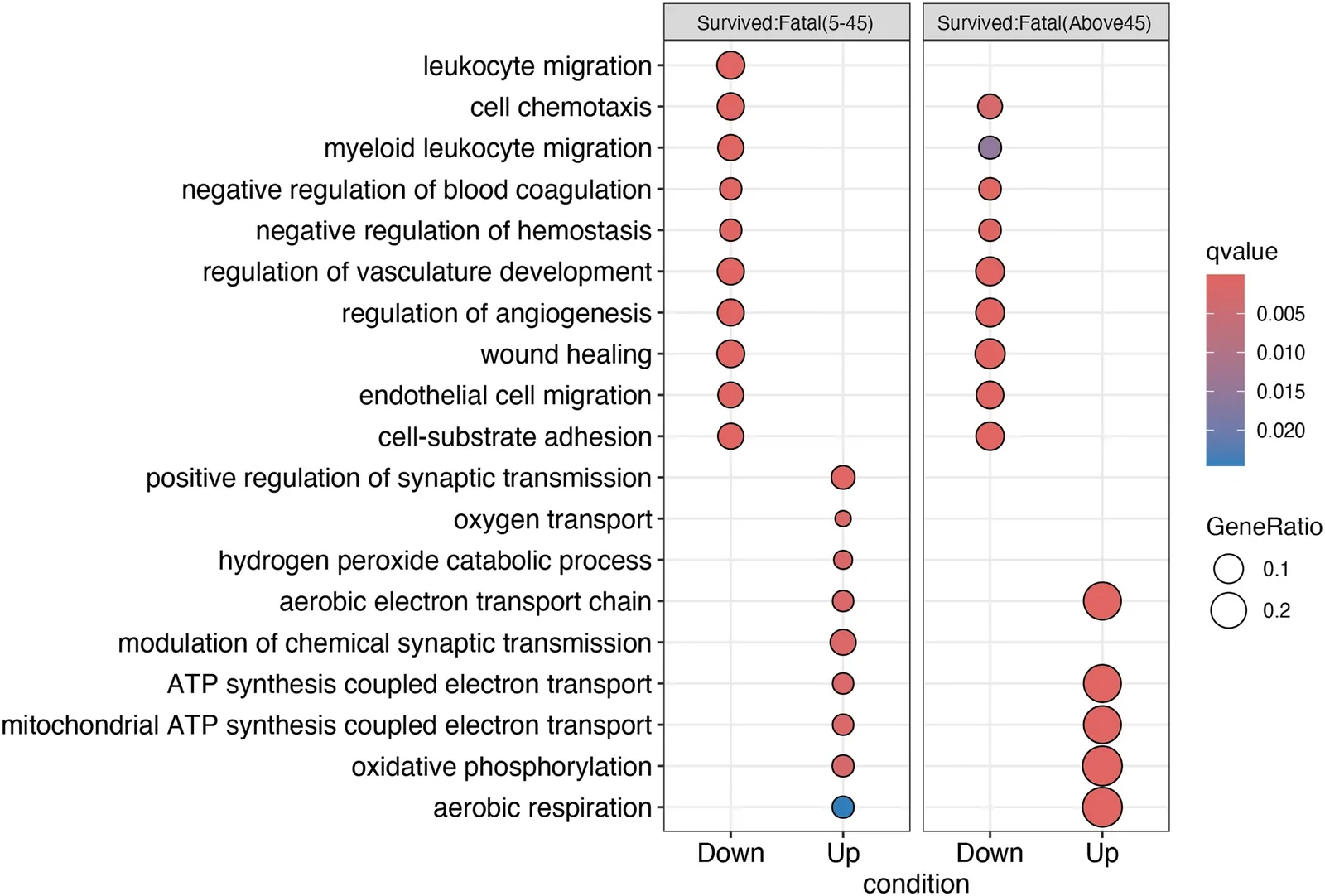
GO enrichment of differentially expressed genes between hospitalized survivors and fatal cases in the age groups of 5–45 and >45 years. Differences in GeneRatio and *q* values are visualized by size and color scale. No significant results were found in the 0- to 4-year age group, so this was omitted from the figure. GO terms with *q* values <.05 are shown. GO, Gene Ontology.

Differences in gene transcription between hospitalized survivors and fatal cases among female and male patients were then investigated without considering age information. A total of 461 and 729 genes were differentially expressed between hospitalized survivors and fatal cases in females and males, respectively. GO enrichment analysis showed similar patterns of enriched functional pathways in females and males, except for the humoral immune response (Figure 3). Although the GO term of humoral immune response was categorized as “general” (ie, level 3), it was grouped separately from other clusters (Supplementary Table 2, Supplementary Figure 4). This analysis confirmed that immune responses were downregulated in hospitalized survivors, regardless of the sex of the patient.

**Figure 3.**
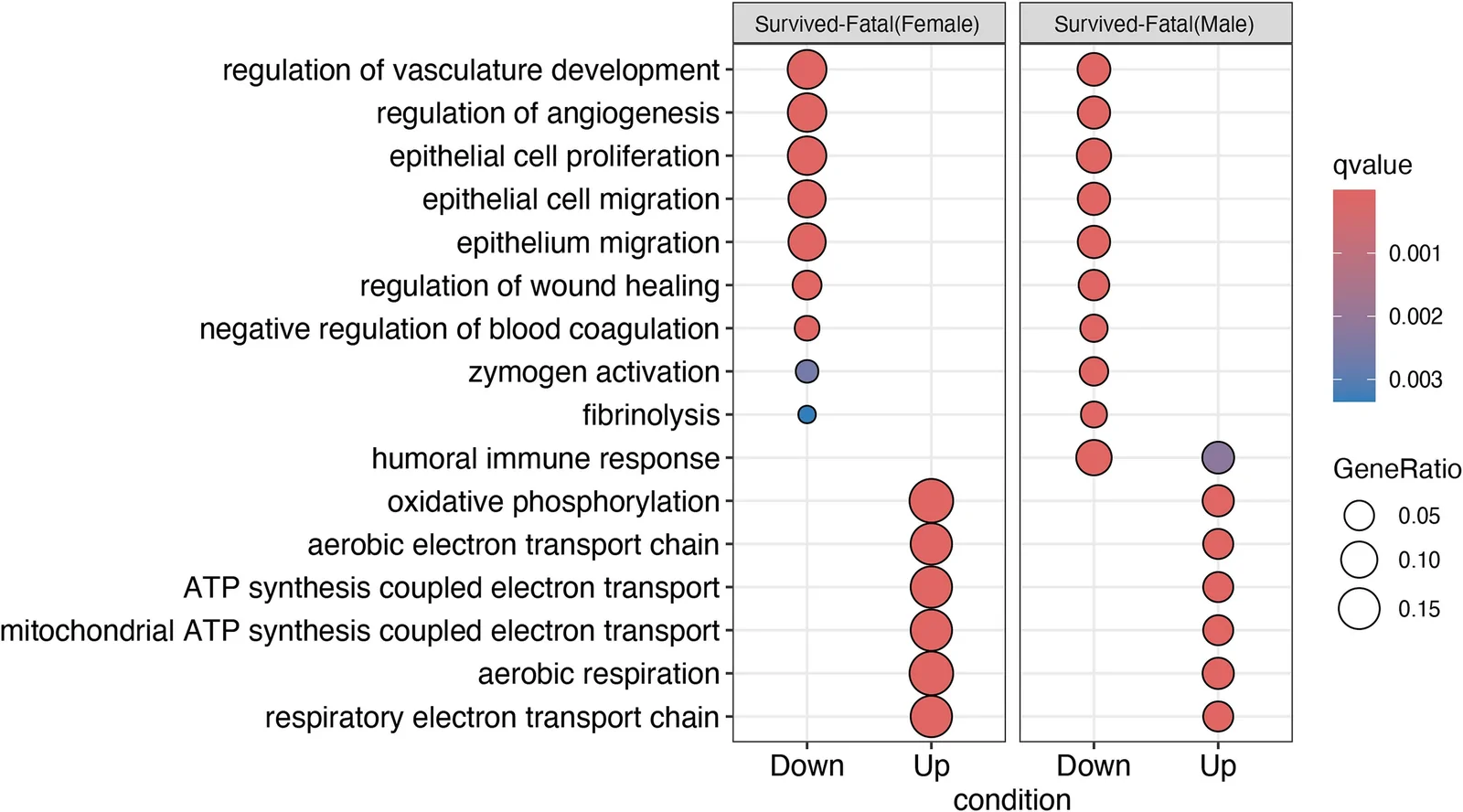
GO enrichment of differentially expressed genes between hospitalized survivors and fatal cases in females and males. Differences in GeneRatio and *q* values are visualized by size and color scale. GO terms with *q* values <.05 are shown. GO, Gene Ontology.

Some of the DGEs were located on the X chromosome in females and males, but none were from the Y chromosome (Supplementary Table 3). Interestingly, these DGEs on the X chromosome were not shared between female and male patients. The gene expression from the X chromosome in male patients was different from that in female patients during EBOV infection, at least in cells present in the blood. When the sex of the patient was factored into the DGE analysis, the GO enrichment pattern of males >45 years old differed from that of females aged 5–45, females >45, and males 5–45 in terms of innate immune response (Figure 4, Supplementary Figure 5).

**Figure 4.**
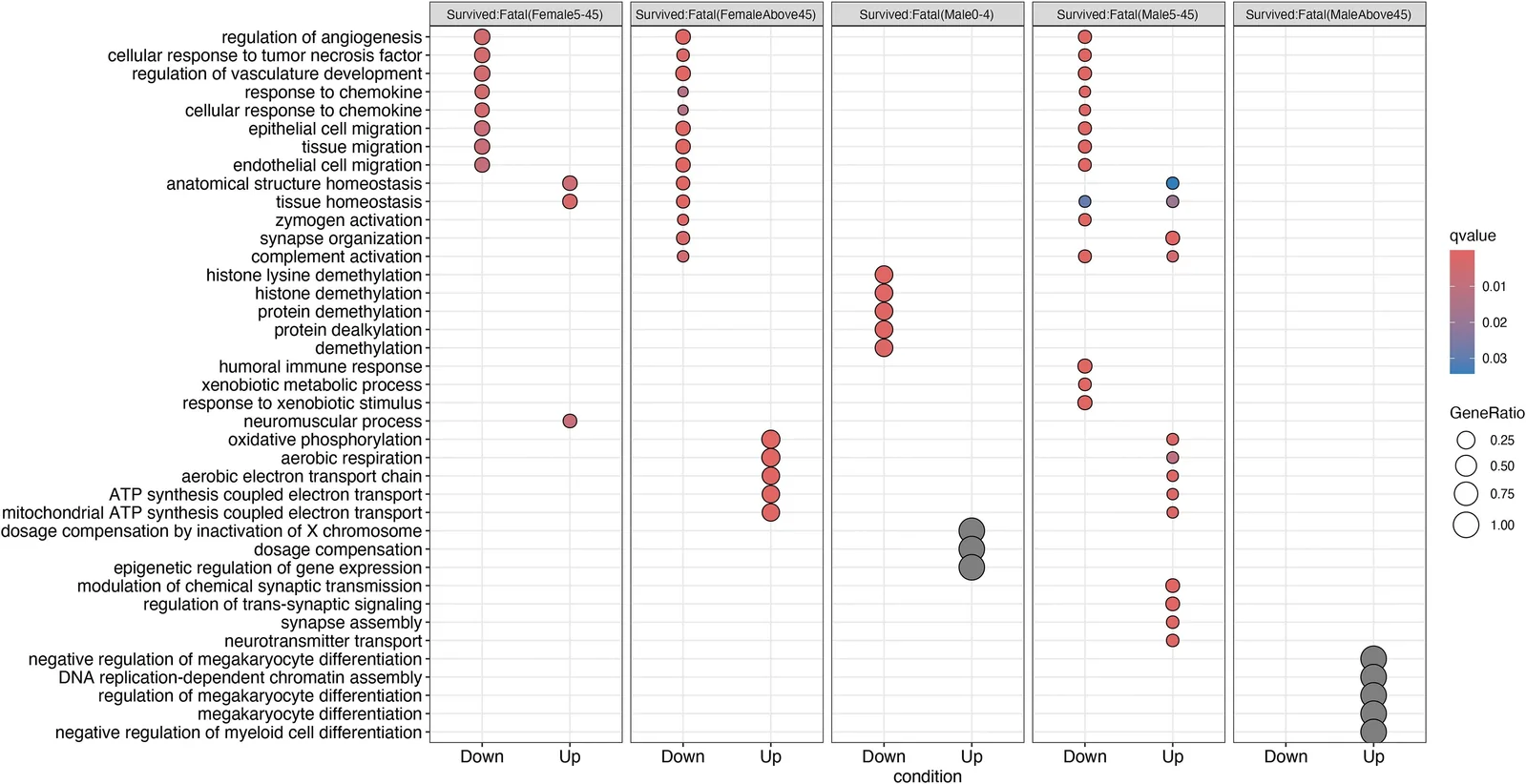
GO enrichment of differentially expressed genes between hospitalized survivors and fatal cases in female and male patients. Differences in GeneRatio and *q* values are visualized by size and color scale. GO terms with *q* values <.05 are shown. No significant results were found in the female group aged 0 to 4 years, so this was omitted from the figure. GO, Gene Ontology.

To investigate whether there was a difference between any age group of hospitalized survivors and fatal cases, regardless of sex, a matrix model design was developed: (survived age group 1 – fatal age group 1) – (survived age group 2 – fatal age group 2). GO enrichment analysis of the data revealed that no GO terms were enriched by the DGEs between the 0–4 and 5–45 groups or between the 0–4 and >45 groups. However, genes upregulated in the >45 group as compared with the 5–45 group were significantly enriched in the cluster related to the response to steroid hormone (Supplementary Figures 6 and 7). This suggests that there was a stronger steroid hormone stimulus in the survivors from the >45 group as compared with the survivors from the 5–45 group. Further analysis revealed a different GO enrichment pattern when sex was included as a parameter (Supplementary Figures 8 and 9).

### Weighted Correlation Network Analysis

The patterns of gene association with given traits were investigated by WGCNA. Highly correlated genes were clustered into the same module based on their expression levels, with modules represented by different colors (Figure 5). WGCNA was used to explore the relationships between these modules, including the genes within them and the age, sex, and viral load of patients with EVD. As can be observed by the depth of color in the first column, these data indicated that the host response was not related to sex in hospitalized survivors but to sex in hospitalized fatal cases. The modules that were red (virus defense), yellow (toll-like receptor 2 signaling pathway/regulation of leukocyte chemotaxis), and magenta (regulation of viral process) had a significant negative association with sex (*P* < .05; downregulated in females vs males) in the hospitalized deceased cases.

**Figure 5.**
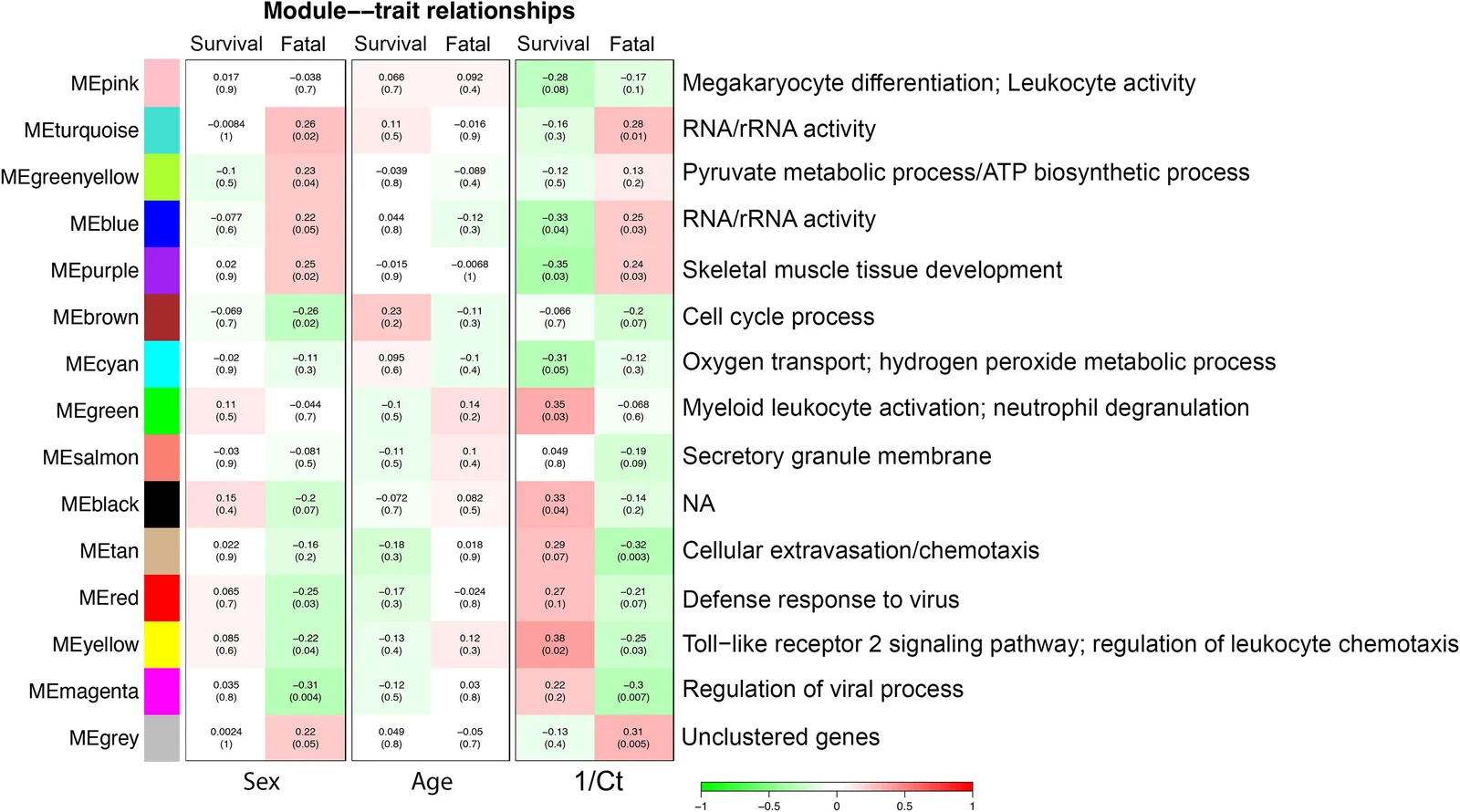
WGCNA for blood samples. Each color (row) corresponds to a module, with the gray module including all unassigned genes. Each column corresponds to a trait (sex, age, viral load). Each cell contains the correlation strength, whether negatively (green) or positively (red) related to the traits, with the *P* value in parentheses. *P* < .05 was considered statistically significant. 1/Ct was used to measure the viral load in each sample. WGCNA linked the biological correlation to the known traits. Ct, cycle threshold; NA, not available; WGCNA, weighted correlation network analysis.

No modules were correlated with age in either hospitalized survivors or fatal cases; genes within the modules that were significantly correlated with age (*P* < .05) were further analyzed (Supplementary Table 4). GO enrichment analysis found that genes involved in lymphocyte differentiation were negatively correlated with age in hospitalized fatal cases but positively correlated with age in hospitalized survivors (Figure 6). Genes driving the significance of the effect on lymphocyte differentiation were partially shared with that of mononuclear cell differentiation in survivors (Supplementary Table 4, Supplementary Figure 10). Within the lymphocyte differentiation pathway, the genes were different to those driving the effect seen in hospitalized fatal cases (Supplementary Figure 10, Figure 6). The gene names and *P* value/*q* value in the enriched GO pathways are summarized in Supplementary Table 5.

**Figure 6.**
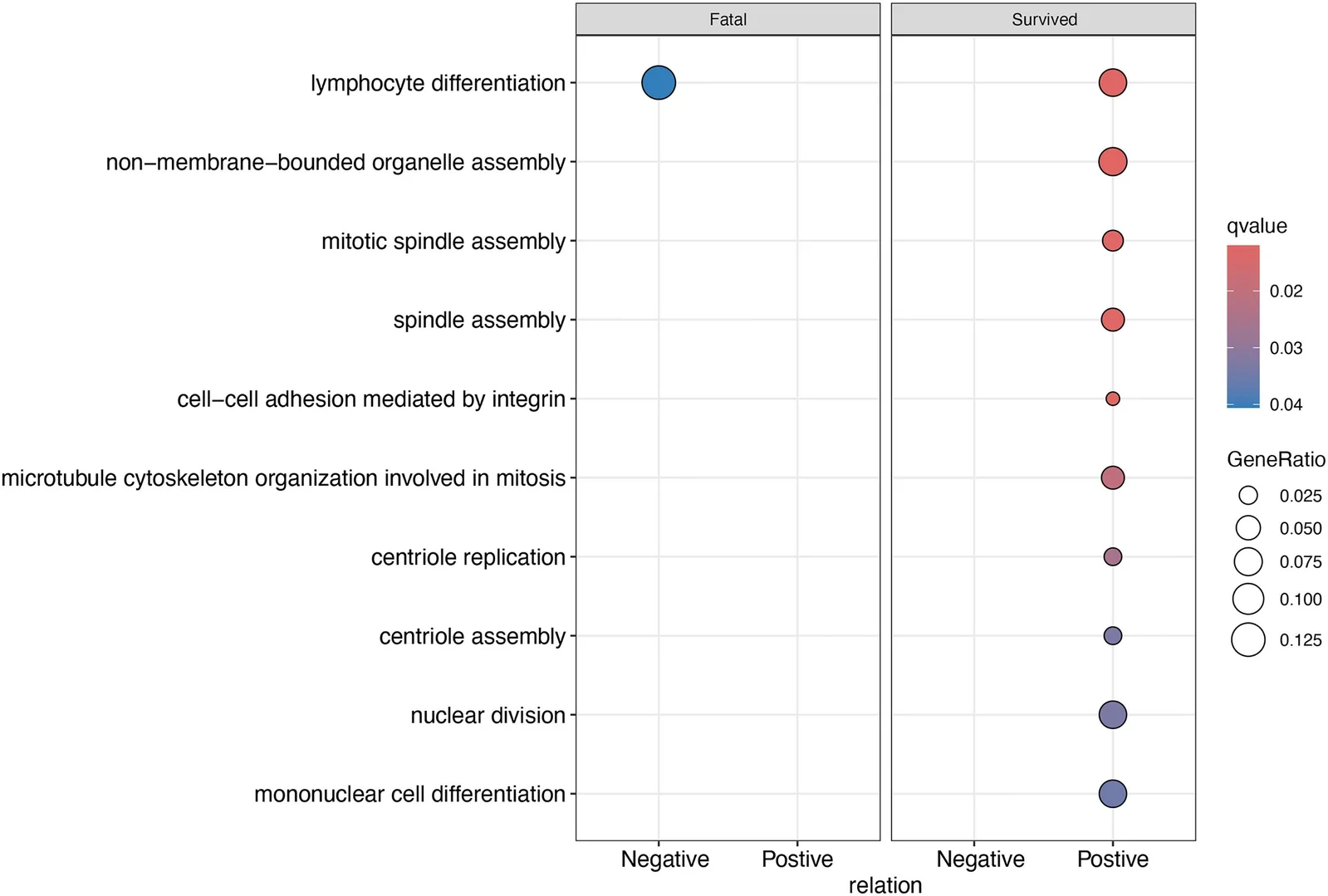
GO enrichment of genes in the modules significantly correlated to the age (*q* < .05) of hospitalized survivors and fatal cases in female and male patients. “Negative” indicates that the genes were negatively related to age increases. “Positive” indicates that the genes were positively related to age increases. Differences in GeneRatio and *q* values are visualized by size and color scale. GO, Gene Ontology.

Several modules—including the toll-like receptor 2 signaling pathway (regulation of leukocyte chemotaxis), myeloid leukocyte activation (neutrophil degranulation), defense response to virus, and regulation of viral processes (MEyellow, MEgreen, MEred, and MEmagenta in Figure 5)—were positively correlated with viral load in hospitalized survivors, although only 2 of these pathways reached statistical significance. In contrast, the opposite correlation was observed in hospitalized fatal cases, although MEgreen and MEred did not reach statistical significance. This suggested that the expression patterns of genes within these modules were linked to a survival phenotype. The hub genes are the central controlled genes located in the module; these can be candidate biomarkers or potential therapeutic targets [22]. Hub genes for all modules, including these 3 modules, were identified and are listed in Supplementary Table 6.

### Viral Population Genetics Varied by Age Group in Male and Female Patients

Viral load and population genetics have been shown to play a role in the outcome of EBOV infection in humans and animal models of the disease [1, 2, 4, 14]. A minor variation of a viral genome is defined as the nondominant nucleic acid sequence at a specific site in a virus genome. To assess the potential effect of sex on minor variants, the average frequencies of minor transitions (Tv), transversions (Ts), synonymous substitutions, and nonsynonymous substitutions at nucleic acid sites on the EBOV genome were compared between the hospitalized survivor group and the hospitalized fatality group (Figure 7). No significant differences in these frequencies in EBOV were observed whether the virus was sampled from male or female patients with the same outcomes, except for nonsynonymous substitutions in the nucleoprotein and viral protein 40 (Supplementary Figure 11). In general, hospitalized survivors had significantly higher minor variation frequencies, except for Tv and nonsynonymous substitutions in males (Figure 7*B* and 7*D*). Further analysis of synonymous and nonsynonymous substitutions in each EBOV gene (Supplementary Figure 11) revealed gene-biased substitutions across different outcomes. Synonymous substitutions were significantly higher in nucleoprotein, glycoprotein, viral protein 30, and RNA-dependent RNA polymerase (L) for female hospitalized survivors and in viral protein 40 and glycoprotein for male hospitalized survivors.

**Figure 7.**
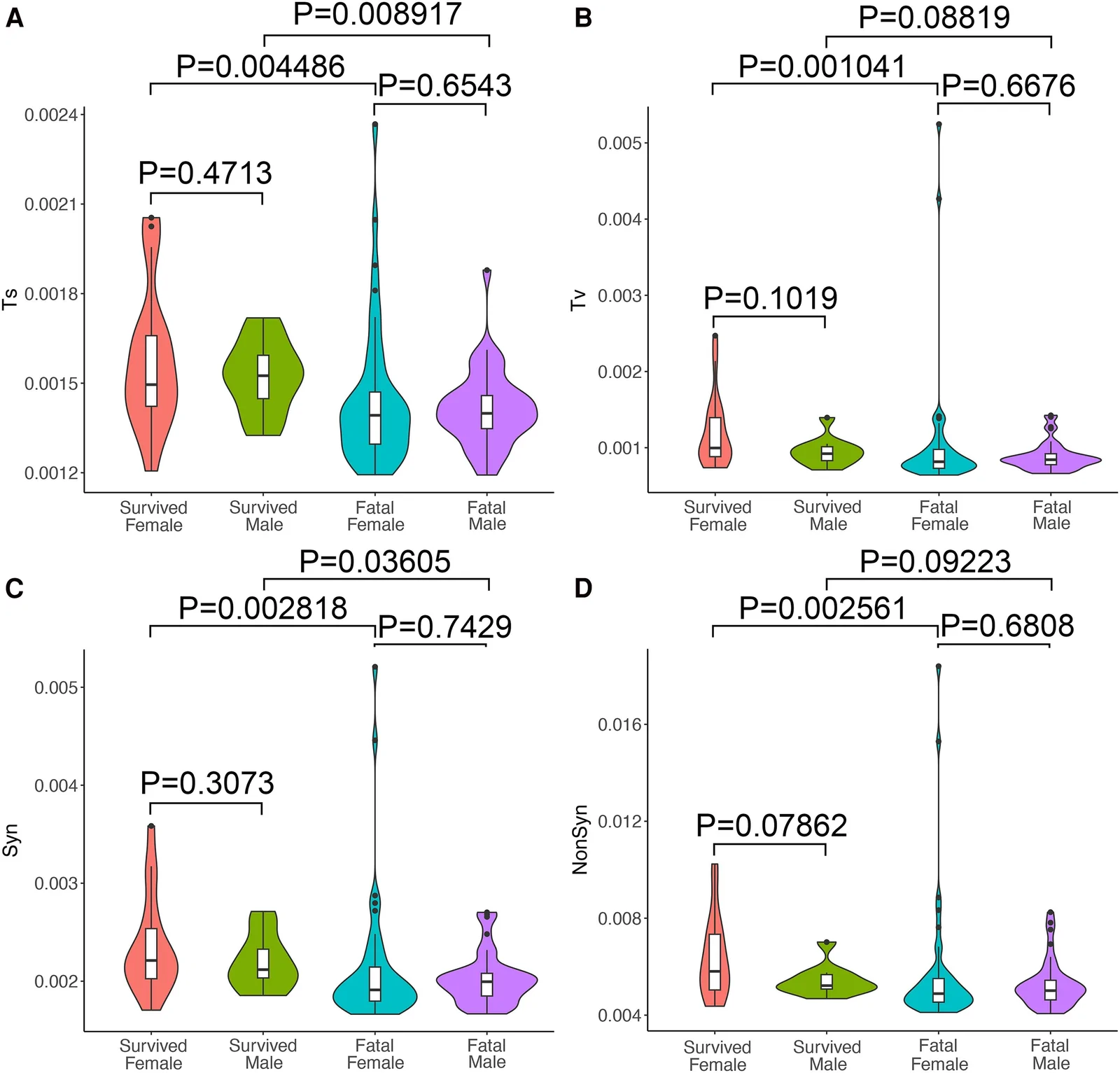
*A–D*, Comparison of the average minor transition (Ts), transversion (Tv), synonymous substitution (Syn), and nonsynonymous substitution (NonSyn) frequencies in hospitalized fatal vs survived cases. *P* values were calculated with a 1-sided Wilcoxon rank sum test as the data did not fit a normal distribution. Data are presented as median (line), interquartile range (box), standard deviation (error bars), filled area of the violin plots (shading), and outliers (circles).

The minor Ts and Tv frequencies were further investigated using a 200-nucleotide sliding window along the EBOV genome. Due to the major clustering of point mutations and resulting regional bias, this sliding window allowed frequency to be calculated across the EBOV genome and compared between groups. Ts and Tv frequencies of hospitalized males who survived or died in the same sliding window were plotted against those of female patients (Supplementary Figure 12A). A datapoint (window) near the reference line (slope = 1 and intercept = 0) indicated that the corresponding hospitalized male and female patients who survived or died had similar Ts or Tv frequencies in that sliding window. In general, the hospitalized survival and fatal groups had more windows with higher Ts than Tv frequencies, but the Ts and Tv frequencies were more diverse between hospitalized females and males who survived. All datapoints (windows) representing Ts frequencies of hospitalized females and males who died clustered closely around the reference line, except for 1 window located between 14 000 and 14 200 on the EBOV genome (Supplementary Figure 12B). This window corresponds to part of the coding region of gene L (amino acids 808–874). Although the average Tv frequencies were not statistically significantly different between hospitalized females and males who died (Figure 7*B*), almost all dots (windows) showed higher Tv frequencies in hospitalized females who died (Supplementary Figure 12B).

## DISCUSSION

Transcriptomic and/or proteomic analysis of blood samples from patients acutely ill with EVD identified differential pathways of disease between those who survived infection and those who died [5, 24, 25]. In this study, the activation of myeloid leukocytes and their chemotaxis were upregulated in hospitalized survivors as compared with fatal cases (Figure 5). This supported previous research reporting dysregulation of inflammation in patients with EVD [6, 26, 27], thus highlighting the importance of the careful balance required in the immune response related to patient outcome in individuals infected with EBOV.

In this study, samples were collected from patients arriving at the treatment unit with varying viral loads, disease severity levels, nutritional deficiencies, and coinfections with diverse bacteria and other viruses [28]. These factors—particularly viral load and disease severity in EVD, which are directly correlated—can cause within-group biological variation (ie, variation among replicates within the same sample group), potentially reducing statistical power in the DGE analysis. However, such within-group biological variations are difficult to avoid when using clinical samples. To mitigate this, the edgeR tool used for DGE analysis can identify true biological differences between compared conditions by distinguishing biological differences from noise (within-group variation) using a negative binomial model. This can function with even a small number of replicates [29]. In addition, our correlation analysis showed that viral load was not associated with sex or age, suggesting that sex- and age-related gene expression changes are independent of viral load. The host responses, pathophysiology, and directionality of EVD are discussed in the Supplementary Text.

In this study, we investigated potential age- and sex-associated differences in the blood transcriptome between male and female patients who were acutely ill with differing outcomes. At a global level, there were no obvious differences in the gene expression profiles in the blood between male and female patients and correlation with outcome, apart from the children in the 0–4 age group (Figure 1, length of line on the x-axis). This may indicate differences between the sexes in the response to EVD at an early age. However, we note the small sample size, as this age group is only one fatal case. This will obviously caution interpretation and conclusions that can be drawn.

Potential differences between male and female patients and the relationship with age were further explored by WGCNA (Figure 5). Genes associated with the lymphocyte differentiation pathway decreased in expression level with age in hospitalized fatal cases. However, this was the opposite in hospitalized survivors (Figure 6), suggesting that the expression level of genes in this pathway is important in determining survival. Genes in this pathway could be targeted for upregulation as part of an immunomodulatory treatment strategy to enhance survival in patients.

EBOV population genetics was examined between male and female patients given the fact that viral population genetics may influence disease [14]. These data indicated that in males in the hospitalized survivor group, the average Ts frequency and nonsynonymous changes in EBOV were significantly higher than in the hospitalized fatality group. But there was no difference in the Tv frequency and nonsynonymous changes in males when patient outcome was considered (Supplementary Figure 12). In general, the pattern of viral population genetics seems to be more variable in the hospitalized survivor group as compared with the hospitalized fatal group. This suggests that more errors in viral replication or RNA modification through cellular processes may occur in females as compared with males and in survivors as compared with fatal cases, potentially contributing to lower functional viral loads. However, this observation could be associated with the cohort of patients analyzed in this study and not generally reflective of the wider population that had EVD. This pattern is consistent with previous findings reporting a lower CFR in female patients with EVD as compared with males, which could be due to reduced viral fitness and/or better host control of infection [13], although there is ambiguity in whether there is a difference in CFR between males and females. We note that differences in viral loads between males and females have been described in human challenge studies of influenza virus [30] and animal models [31]. There may be a relationship between mutational accumulation and viral load or vice versa.

Generally, there is an emerging picture that animal models should be appropriately tailored to the disease being studied. For example, with SARS-CoV-2 and influenza virus, severe disease correlates with age and comorbidities. However, sometimes through necessity and availability, in vivo experiments use younger animals, which tend to have no comorbidities. Obese ferrets more closely model severe influenza virus infection than younger and presumably healthier ferrets [32], and obesity has an impact on genetic variation in the virus [33]. In a ferret model of EVD, there was no difference between male and female animals [21]. In humans, subtle sex- and age-specific differences exist, and these could be considered in future immune modulatory treatment strategies and in animal models of disease.

## Supplementary Material

jiag305_Supplementary_Data

## Supplementary Data

Supplementary materials are available at *The Journal of Infectious Diseases* online (http://jid.oxfordjournals.org/). Supplementary materials consist of data provided by the author that are published to benefit the reader. The posted materials are not copyedited. The contents of all supplementary data are the sole responsibility of the authors. Questions or messages regarding errors should be addressed to the author.
