# Supplementary material for: Molecular Phenotypes of Sex- and Age-Specific Differences Relating to Outcome in Patients With Ebola Virus Disease
Source: J Infect Dis. 2026 Jun 17;234(4):e838–47. doi: 10.1093/infdis/jiag305 (PMC13668345; doi:10.1093/infdis/jiag305)
Supplement: Supplementary file 1 [file jiag305_supplementary_data.zip › Supplementary Text.docx]

**Host responses and Pathophysiology**

Ebola virus disease (EVD) is known to induce uncontrolled pro-inflammatory cytokine responses, known as a “cytokine storm,” which have been linked to vascular impairment, disseminated intravascular coagulation, and massive loss of innate and adaptive immune cells [2, 3].

In this study, GO enrichment analysis showed that immune response and wound-healing pathways were downregulated in hospitalised survivors compared with hospitalised fatal cases (Figure 2). This observation is consistent with the known pathophysiology of EVD. In humans and nonhuman primates, fatal cases are typically associated with higher viral loads, leading to uncontrolled innate immune activation and multi-organ damage [3, 4]. As a result of widespread tissue injury, wound-healing and tissue-repair programmes are aberrantly activated. Therefore, the increased expression of immune and wound-healing genes observed in fatal cases likely reflects ongoing tissue damage and uncontrolled viral replication rather than effective host defence. In contrast, survivors appear to downregulate these pathways once viral replication is controlled, thereby avoiding immune-mediated damage.

When comparing hospitalised survivors with hospitalised fatal cases in the youngest age group, no enrichment of innate immune response or wound-healing GO pathways was observed after adjusting for sex (Figure 4). Specifically, these pathways were not enriched in the 0-4 year age group. This suggests that survival in young patients is not driven by enhanced antiviral immunity. Although these results may be limited by the small sample size, they are consistent with evidence from coronavirus studies showing that children generally experience cytokine storms less frequently and less severely than adults, likely due to their less mature immune systems [5, 6].

In this study, weighted gene co-expression network analysis (WGCNA) was used to investigate sex-based differences in hospitalised survivors and hospitalised fatal cases. This analysis identified gene modules related to Toll-like receptor signalling, myeloid activation, and viral defence/regulation that were negatively correlated with sex (downregulated in females compared to males) only in hospitalised fatal cases (Figure 5). These findings suggest sex-specific differences in the degree of dysregulated innate immunity among fatal cases, whereas a similarly controlled innate immune response is observed in male and female survivors.

**Directionality (death/survival)**

Ebola has affected young children most severely, killing around 90 per cent of children aged under a year and around 80 per cent of children aged one to four years who are infected. Older children are much more likely to survive the disease – it has killed 52 per cent of children infected aged 10 to 15. For adults aged 16 to 44, the case fatality rate is 65 per cent, and those aged 45 or over, where it was 78.7% [7]. Although, we did not specially analyse the cases aged under a year, as discussed above we found survival in 0-4 group is not driven by enhanced antiviral immunity.

Cases of EVD (2013–2015) showed that female patients were significantly less likely to die than male patients. Overall, female cases had a significantly lower case fatality rate than male cases. This survival difference remained significant across all 5-year age intervals [8]. GO enrichment analysis revealed similar patterns of enriched functional pathways in females and males, except for the humoral immune response (Figure 3). The humoral immune response is a key branch of the adaptive immune system that defends against extracellular pathogens by producing antigen-specific antibodies. The humoral immune response has also been suggested to contribute to immunity against EBOV infection in nonhuman primates vaccinated with AdHu5 expressing GP [9].  Differentially expressed genes enriched in this GO pathway may be relevant to the observed survival differences between males and females.

**References**

1.	Rosati, D., et al., *Differential gene expression analysis pipelines and bioinformatic tools for the identification of specific biomarkers: A review.* Comput Struct Biotechnol J, 2024. **23**: p. 1154–1168.

2.	Yamaoka, S. and H. Ebihara, *Pathogenicity and Virulence of Ebolaviruses with Species-and Variant-specificity.* Virulence, 2021. **12**(1): p. 885–901.

3.	Colavita, F., et al., *Inflammatory and humoral immune response during Ebola virus infection in survivor and fatal cases occurred in Sierra Leone during the 2014–2016 outbreak in West Africa.* Viruses, 2019. **11**(4): p. 373.

4.	Reynard, S., et al., *Early control of viral load by favipiravir promotes survival to Ebola virus challenge and prevents cytokine storm in non-human primates.* PLoS Neglected Tropical Diseases, 2021. **15**(3): p. e0009300.

5.	Sinaei, R., et al., *Why COVID-19 is less frequent and severe in children: a narrative review.* World Journal of Pediatrics, 2021. **17**(1): p. 10–20.

6.	Luo, C., et al., *The mechanisms of milder clinical symptoms of COVID-19 in children compared to adults.* Italian Journal of Pediatrics, 2024. **50**(1): p. 28.

7.	Gulland, A., *Ebola mortality is highest among babies, finds study.* BMJ, 2015. **350**.

8.	Team, W.E.R., *Ebola virus disease among male and female persons in West Africa.* New England Journal of Medicine, 2016. **374**(1): p. 96–98.

9.	Wong, G., et al., *Immune parameters correlate with protection against ebola virus infection in rodents and nonhuman primates.* Science translational medicine, 2012. **4**(158): p. 158ra146–158ra146.
