# Supplementary material for: Molecular Phenotypes of Sex- and Age-Specific Differences Relating to Outcome in Patients With Ebola Virus Disease
Source: J Infect Dis. 2026 Jun 17;234(4):e838–47. doi: 10.1093/infdis/jiag305 (PMC13668345; doi:10.1093/infdis/jiag305)
Supplement: Supplementary file 1 [file jiag305_supplementary_data.zip › supplementary_figures.docx]

*Figure S1. Data analysis flowchart in this study.*

*Figure S2. Correlation between* *viral load (Ct value) and Age and Sex. A Spearman rank correlation test was used to estimate the correlations. The solid line represents the line of regression, including a 95% confidence region (light grey area).*

*Figure S3. Enrichment map organizes enriched terms in Figure 2 into a network with edges connecting by overlapping gene sets measured by* *Jaccard index.*

*Figure S4. Enrichment map organizes enriched terms in Figure 3 into a network with edges connecting by overlapping gene sets measured by Jaccard index.*

*Figure S5.* *Enrichment map organizes enriched terms in Figure 4 into a network with edges connecting by overlapping gene sets measured by Jaccard index.*

*Figure S6. GO enrichment of differentially expressed genes between hospitalised survivors and fatal cases of the* 5-45 and above 45 age groups regardless of sex. *Differences in the GeneRatio and q values are visualized using size and colour scale.* *GO terms with* *q values <0.05 were showed. The comparisons without enriched GO terms were not shown*

*Figure S7. Enrichment map organizes enriched terms in Figure 4 into a network with edges connecting by overlapping gene sets measured by Jaccard index.*

*Figure S8. GO enrichment of differentially expressed genes between hospitalised survivors and fatal cases of the 0-4,* 5-45 and above 45 age groups regarding sex. *Differences in the GeneRatio and q values are visualized using size and colour scale.* *The comparisons without enriched GO terms were not showed. GO terms with q values <0.05 were showed.*

*Figure S9. Enrichment map organizes enriched terms in Figure S8 into a network with edges connecting by overlapping gene sets measured by Jaccard index.*

*Figure S10. Enrichment map organizes enriched terms in Figure 6 into a network with edges connecting by overlapping gene sets measured by Jaccard index.*

*Figure S11.* *Comparison of the* *synonymous substitution and non-synonymous* *substitution frequencies for each EBOV genes in hospitalised fatal versus hospitalised survived cases. The p-values were calculated with one-sided Wilcoxon rank sum test as the data did not fit a normal distribution.*

*Figure S12. Ts (blue) and Tv (orange) frequencies of hospitalised survived (A) or fatal (B) male patients in the same sliding window were plotted against hospitalised survived (A) or fatal (B) female patients.*
